# A sheep in wolf's clothing; a case of renal leiomyoma masquerading as hereditary leiomyomatosis and renal cell carcinoma

**DOI:** 10.1016/j.eucr.2023.102567

**Published:** 2023-09-22

**Authors:** Nicole Uzzo, Matthew Loecher, Robert G. Uzzo, Daniel D. Eun

**Affiliations:** a Fox Chase Cancer Institute, Philadelphia, PA, USA; b Temple University Hospital, Philadelphia, PA, USA

**Keywords:** Leiomyoma, Renal mass, Pregnancy, Robotics

## Abstract

Active surveillance has become a standard of care for the management of small renal masses. Decision to transition from surveillance to intervention relies on several factors including growth kinetics, histologic grade on biopsy and patient comorbidities. Management of renal masses in pregnancy presents a unique change when clinical triggers must be weighed with risk to fetus. We present the case of a third trimester patient with an enlarging and enhancing renal mass managed with robotic assisted laparoscopic partial nephrectomy. Histologic analysis was consistent with renal leiomyoma. Renal leiomyomas are a rare benign mesenchymal tumor influenced by changes in progesterone-estrogen axis.

## 1 Introduction

Mesenchymal tumors are a rare phenomenon in the urinary tract with less than a hundred renal leiomyomas represented in the literature.^1^ With such rarity and imaging features similar to malignant renal masses their diagnosis remains challenging.^2^ The majority of these lesions are present in women with only a third of cases reported in men.^3^ Diagnosis is most common between the second and the fifth decades of life often incidentally during the work up of other complaints. Several case reports however have demonstrated symptomatic bleeding and flank pain. We report the case of renal leiomyoma in a 40-year-old pregnant female found to have a small renal mass prior to pregnancy and during active surveillance was found to have rapid growth of her lesion triggering intervention with robotic assisted laparoscopic partial nephrectomy.

## 2 Case description

**Fig. 1:**
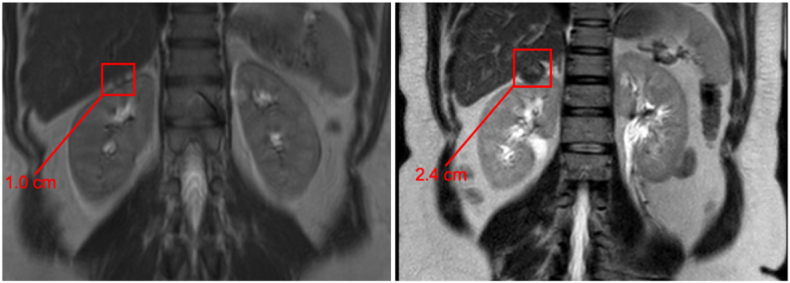
T2 weighted coronal MRI images of right renal mass on presentation (right) and six months prior (left).

A 40-year-old woman with history uterine fibroids status post laparoscopic myomectomy at age 30 initially presented to primary care physician for evaluation of abdominal pain. Abdominal imaging was obtained (Fig. 1.) with and MRI of the abdomen with and without contrast which showed a 1.0 cm enhancing right upper pole renal mass as well as multiple large uterine fibroids. She was referred to urology for consultation and recommended to undergo active surveillance with repeat imaging six months after her MRI. Further work up at that time revealed normal hemoglobin and hematocrit, an eGFR of 104 and no microscopic hematuria. She excitedly became pregnant between her interval imaging and an abdominal ultrasound was obtained at her six month follow up which showed interval increase to 2.0 cm. Cross sectional imaging was ordered to confirm growth kinetics and a follow up MRI seven months after her initial imaging showed a 2.3 cm enhancing lesion (Fig. 1.).

With a more than doubling in size over approximately 6 months and history of uterine fibroids the patient was counseled that this may suggest an HLRCC mutation. She was referred for genetic counseling. Given the risk of metastasis with <3 cm masses she was recommended to undergo partial nephrectomy.

Consultation with her high-risk maternal fetal medicine specialist was performed preoperatively. Intraoperative noninvasive fetal monitoring was performed within the sterile field. Insufflation was obtained in the right upper quadrant most caudad to the gravid uterus. Lateral decubitus position was utilized as in standard partial nephrectomy fashion. The mass was well circumscribed and encapsulated similar to malignant lesions. Her case proceeded without complication with overall operative time of 66 minutes and warm ischemia clamp time of 7 minutes. Her recovery was uncomplicated with no significant change in her post operative hemoglobin or renal function. At forty weeks gestation she underwent a scheduled caesarean section with uncomplicated delivery and healthy fetus.

Gross examination revealed a 2.6 cm well circumscribed mass with microscopic examination showing spindle cells in hyalinized stromal tissue. No mitosis, necrosis, or atypical cells were present to suggest an underlying sarcoma. Immunohistochemical evaluation was positive for desmin, SMA, and vimentin and negative for S100, Melan-A, HMB-45, DOG-1, *C*-Kit (CD117), AE1/AE3, and CD34. Consistent with leiomyoma without malignant features.^4^

## 3 Discussion

We present this patient to highlight the case of a sheep in wolf's clothing. With the benign genotype, renal leiomyoma, presenting as the aggressive phenotype, papillary type II RCC associated with HLRCC. Given their rarity, little data is available to suggest patients with a history of uterine fibroids are at increased risk for leiomyomas outside the uterus. Again, due to lack of prevalence, the association with extra-uterine leiomyomas and hormonal regulation is not clear. However, if biology similar to their uterine counterparts, the American College of Gynecologists has clear guidelines implicating menstrual cycle hormone changes with alterations in fibroid size and symptoms.^5^ One could hypothesize that with delivery her mass may have involuted and decreased in size.

In addition to the natural history of her disease, it is important to highlight the importance of patient centered counseling and risk assessment. This case should stress to urologic oncologists and minimally invasive surgeons that laparoscopy in the second and third trimester is safe, controlled and associated with similar outcomes in non-gravid patients.^6^ If her renal mass and genetic testing were consistent with HLRCC delay in her care until after delivery would pose unnecessary risk to mother.

## 4 Conclusion

Renal leiomyomas are rare and difficult to diagnosis. We present the case of a rapidly enlarging renal mass in a pregnant woman with uterine fibroids found to have a renal leiomyoma after partial nephrectomy during pregnancy. This unique case stresses the importance of balancing risks and benefits as well as a patient centered model of care. We encourage urologists to safely perform laparoscopy when our patients care demands it.

## Declaration of competing interest

No declarations to disclose.
